# Supplementary material for: Community-based conservation with formal protection provides large collateral benefits to Amazonian migratory waterbirds
Source: PLoS One. 2021 Apr 8;16(4):e0250022. doi: 10.1371/journal.pone.0250022 (PMC8031428; doi:10.1371/journal.pone.0250022)
Supplement: S1 Fig — There were no significant ANOVA differences between surveys conducted at 06:30h - 08:00h (Time 1) or 08:30h - 10:00h (Time 2) in any of the surveyed species: (a) Rynchops niger, (b) Phaetusa simplex, (c) Sturnella superciliaris, and (d) Neochen jubata. Time of survey was therefore not included in any subsequent models. (PDF) [file pone.0250022.s001.pdf]

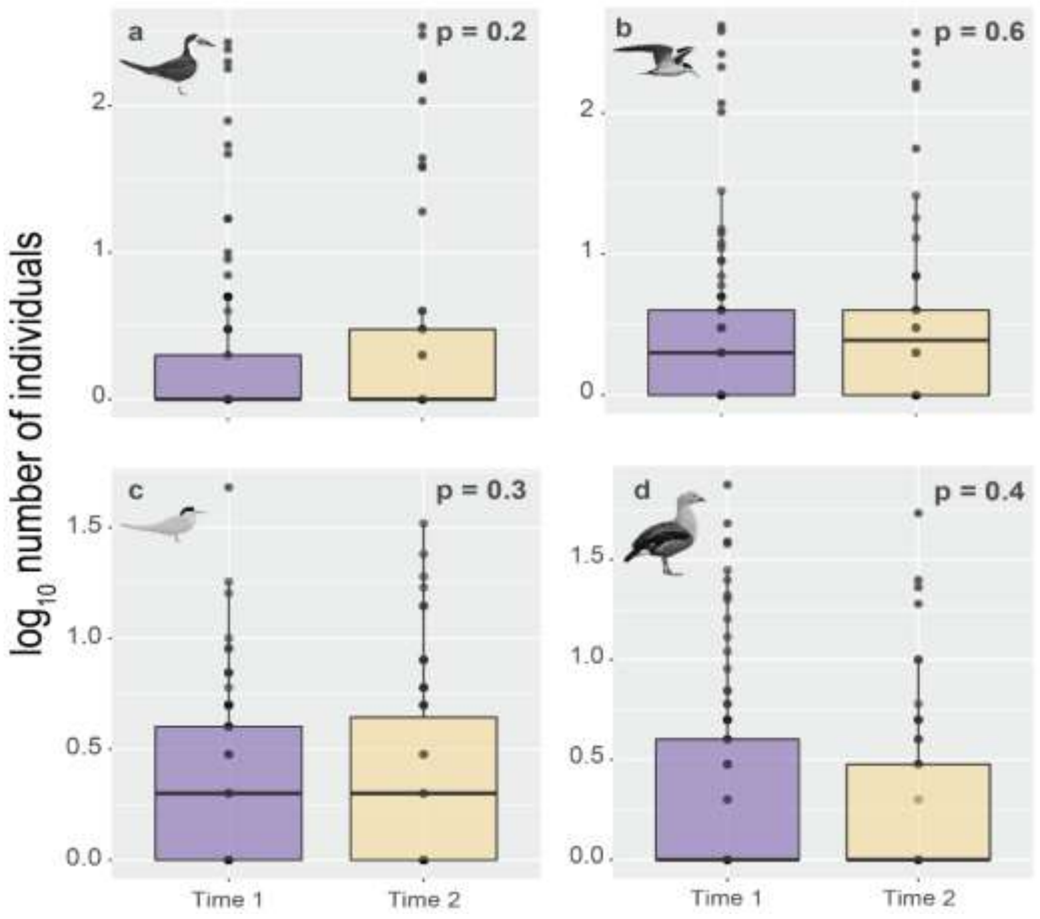

S1 Fig. Testing the effect of time of the day on our surveys of waterbird abundance on fluvial beaches along the Juruá River, western Brazilian Amazonia. There were no significant ANOVA differences between surveys conducted between 06:30h and 08:00h (Time 1) or between 08:30h to 10:00h (Time 2) in any of the surveyed species: (a) *Rynchops niger*, (b) *Phaetusa simplex*, (c) *Sturnella superciliaris,* and d) *Neochen jubata.* Time of survey was therefore not included in any subsequent models.
