# Supplementary material for: Community-based conservation with formal protection provides large collateral benefits to Amazonian migratory waterbirds
Source: PLoS One. 2021 Apr 8;16(4):e0250022. doi: 10.1371/journal.pone.0250022 (PMC8031428; doi:10.1371/journal.pone.0250022)
Supplement: S2 Fig — We used a subset of 28 beaches to test (paired t-test) for interannual differences between our surveys conducted in 2016 (this study) and surveys conducted in 2014 (Campos-Silva et al. 2018). Surveys of population size for (a) Rynchops niger and (b) Phaetusa simplex were consistent at “CBC + PA” and “PA only” beaches in each of these surveys. (PDF) [file pone.0250022.s002.pdf]

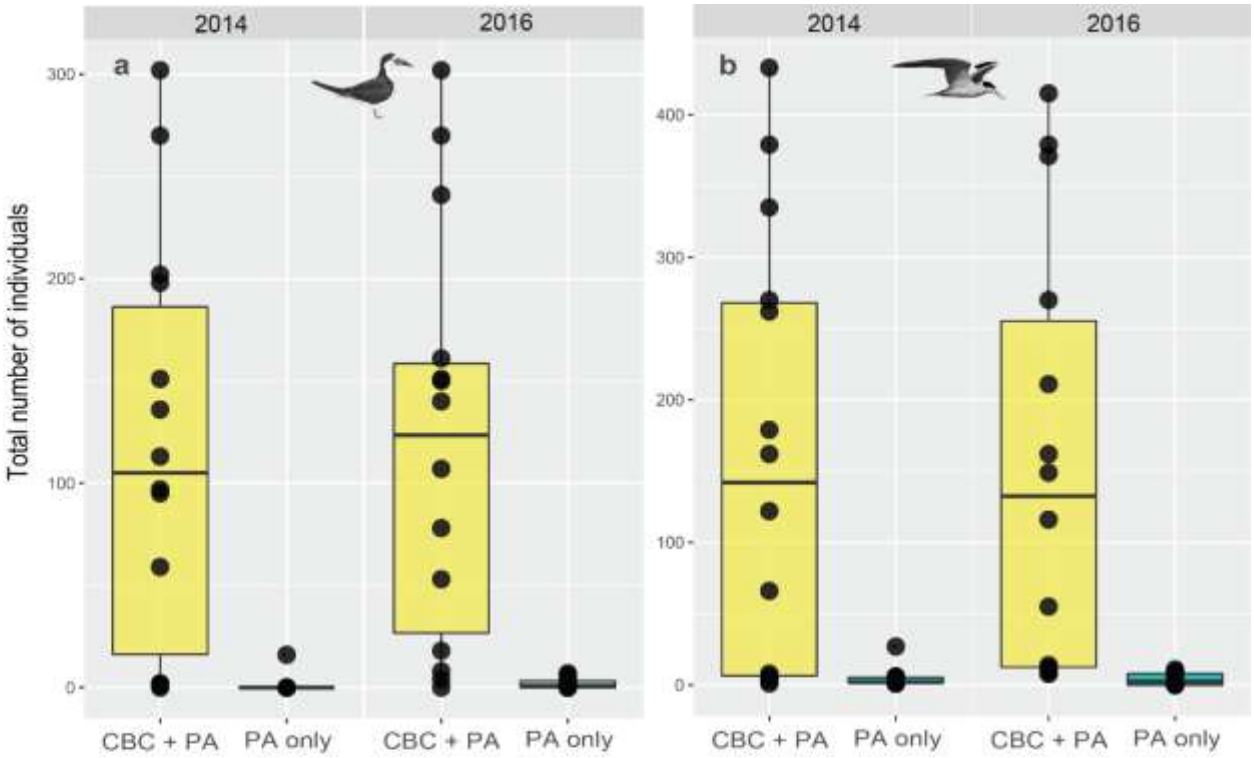

S2 Fig. Testing the effect of year on our surveys of waterbird abundance on fluvial beaches along the Juruá River, western Brazilian Amazonia. We used a subset of 28 beaches to test (paired t-test) for interannual differences between our surveys conducted in 2016 (this study) and surveys conducted in 2014 (Campos-Silva et al. 2018). Surveys of population size for (a) *Rynchops niger* and (b) *Phaetusa simplex* were consistent at "CBC + PA" and "PA only" beaches in each of these surveys.
