# Supplementary material for: Community-based conservation with formal protection provides large collateral benefits to Amazonian migratory waterbirds
Source: PLoS One. 2021 Apr 8;16(4):e0250022. doi: 10.1371/journal.pone.0250022 (PMC8031428; doi:10.1371/journal.pone.0250022)
Supplement: S3 Fig — Population size of target species (a) Rynchops niger, R2 = 0.8, p<0.01; (b) Phaetusa simplex, R2 = 0.6, p<0.01; (c) Sturnella superciliaris, R2 = 0.1, p<0.01; and (d) Neochen jubata, R2 = -0.003, p = 0.4) as a function of the number of years that the beach had been under local community protection for unprotected sites (red), PA-only (blue), CBC-only (orange) and CBC + PA (yellow). Unprotected sites and PA-only were left as zero years of protection, considering that in both categories there is no local site protection by rural communities. (PDF) [file pone.0250022.s003.pdf]

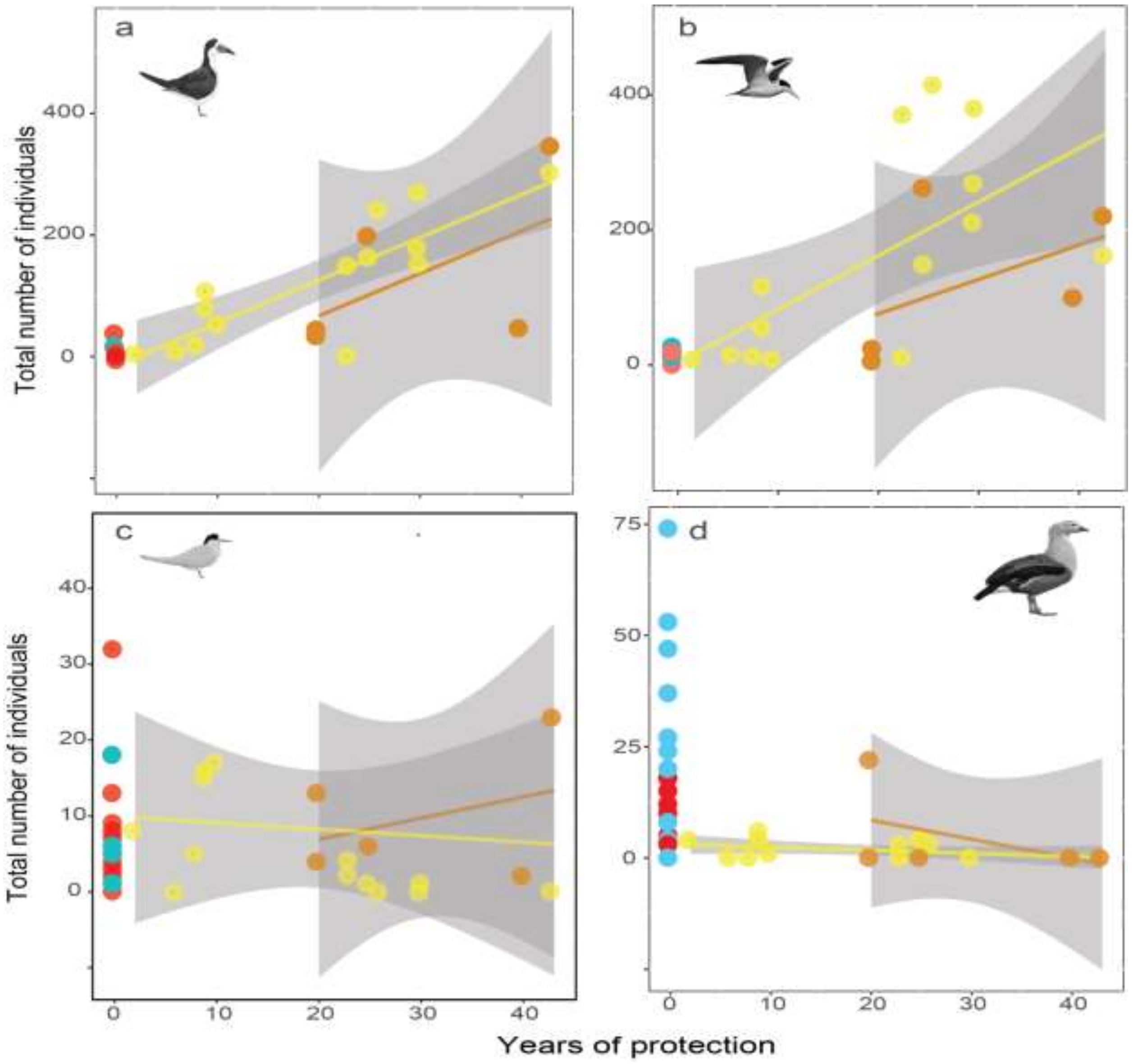

S3 Fig. Testing the effect of protection duration for waterbird abundance on fluvial beaches along the Juruá River, western Brazilian Amazonia. Population size of target species (a) *Rynchops niger*, $R^2 = 0.8$, $p<0.01$; (b) *Phaetusa simplex*, $R^2 = 0.6$, $p<0.01$; (c) *Sturnella superciliaris*, $R^2 = 0.1$, $p<0.01$; and (d) *Neochen jubata*, $R^2=-0.003$, $p=0.4$) as a function of the number of years that the beach had been under local community protection for unprotected sites (red), PA-only (blue), CBC-only (orange) and CBC + PA (yellow). Unprotected sites and PA-only were left as zero years of protection, considering that in both categories there is no local site protection by rural communities.

References

Balestra, RAM. Manejo Conservacionista e Monitoramento Populacional de Quelônios Amazônicos. Instituto Brasileiro do Meio Ambiente e dos Recursos Naturais Renováveis (Ibama), Brasilia. 2016.

Campos-Silva, JV, Hawes, JE, Andrade, PC, Peres, CA. Unintended multispecies co-benefits of an Amazonian community-based conservation programme. Nature Sustainability. 2018; 1(11), 650-656.

Campos-Silva, JV, Hawes, JE, Freitas, CT, Andrade, PC, Peres, CA. Community-Based Management of Amazonian Biodiversity Assets. In: Balduf C. (ed.) Participatory Biodiversity Conservation. Springer, Cham, pp. 99-111. 2020.
