# Supplementary material for: Community-based conservation with formal protection provides large collateral benefits to Amazonian migratory waterbirds
Source: PLoS One. 2021 Apr 8;16(4):e0250022. doi: 10.1371/journal.pone.0250022 (PMC8031428; doi:10.1371/journal.pone.0250022)
Supplement: S1 Table — Full dataset with the number of individuals for each waterbird species per fluvial beach (n = 155), showing protection status, location coordinates, distance to nearest community, distance to nearest town and beach area. (PDF) [file pone.0250022.s004.pdf]

S1 Table. Abundance of four waterbird species surveyed along the Juruá River, western Brazilian Amazonia. Full dataset with the number of individuals for each waterbird species per fluvial beach (n=155), showing protection status, location coordinates, distance to nearest community, distance to nearest town and beach area.

**Beach ID**	**Protection status**	**Lat**	**Long**	**Area (ha)**	**Nearest comm. (km)**	**Nearest town (km)**	***Rynchops niger***	***Phaetusa simplex***	***Sturnella superciliaris***	***Neochen jubata***
1	unprotected	-6.71551	-69.8435	11.0	2.3	10.2	0	0	0	0
2	unprotected	-6.69534	-69.8297	6.0	2.2	4.8	0	1	0	0
3	Unprotected	-6.71523	-69.8231	3.0	0.8	12.1	0	17	4	0
4	Unprotected	-6.70946	-69.8191	10.7	0.6	13.3	0	2	0	0
5	Unprotected	-6.70328	-69.7945	5.2	3.0	15.8	0	1	0	0
6	Unprotected	-6.63195	-69.7844	29.0	6.3	28.0	0	1	1	0
7	Unprotected	-6.58119	-69.7704	25.7	2.1	46.9	0	0	1	0
8	Unprotected	-6.5729	-69.7436	33.1	4.0	44.1	0	0	2	0
9	Unprotected	-6.58424	-69.7341	24.8	4.3	39.5	0	0	2	0
10	Unprotected	-6.55836	-69.7229	24.4	5.7	54.5	2	2	7	2
11	Unprotected	-6.5584	-69.6667	32.0	2.3	60.8	0	2	1	0
12	Unprotected	-6.57262	-69.608	51.0	0.6	87.5	0	0	0	1
13	Unprotected	-6.56225	-69.5811	10.7	3.1	83.6	0	1	2	0
14	Unprotected	-6.55358	-69.5793	39.9	3.1	81.4	0	1	0	0
15	Unprotected	-6.54794	-69.4649	31.5	1.2	116.9	0	3	0	0
16	Unprotected	-6.5285	-69.4402	27.6	3.0	123.9	0	0	3	0
17	Unprotected	-6.55381	-69.4321	55.9	0.6	127.4	0	2	0	0
18	Unprotected	-6.61417	-69.3325	32.3	5.9	158.1	3	1	5	0
19	Unprotected	-6.60819	-69.325	21.0	6.1	160.5	1	2	0	0
20	Unprotected	-6.59564	-69.292	39.5	8.3	169.9	2	1	0	9
21	Unprotected	-6.60639	-69.2899	20.0	8.8	171.4	2	1	0	0
22	Unprotected	-6.61022	-69.1406	33.8	5.1	197.0	0	0	6	6
23	Unprotected	-6.52919	-69.1228	56.7	9.8	211.4	2	3	2	5

Beach ID	Protection status	Lat	Long	Area (ha)	Nearest comm. (km)	Nearest town (km)	*Rynchops niger*	*Phaetusa simplex*	*Sturnella superciliaris*	*Neochen jubata*
24	Unprotected	-6.52075	-69.1066	32.7	9.6	215.5	1	0	1	5
25	Unprotected	-6.53658	-68.9823	14.1	4.3	241.4	0	0	0	0
26	Unprotected	-6.5651	-68.9796	11.5	5.5	230.8	0	0	1	2
27	Unprotected	-6.54688	-68.9736	14.1	5.5	229.1	0	0	0	2
28	Unprotected	-6.53649	-68.948	19.4	6.6	225.6	0	1	3	4
29	Unprotected	-6.55414	-68.9277	13.7	2.9	214.3	6	1	3	4
30	Unprotected	-6.53567	-68.9179	13.7	5.1	208.5	0	3	4	2
31	Unprotected	-6.5294	-68.8955	9.8	6.2	189.4	4	0	0	0
32	Unprotected	-6.52297	-68.8214	37.1	2.0	181.1	38	2	5	9
33	Unprotected	-6.5188	-68.8064	20.1	0.6	178.3	0	0	0	1
34	Unprotected	-6.48576	-68.7996	20.6	1.0	171.8	0	1	2	0
35	Unprotected	-6.50604	-68.7962	1.3	2.3	175.4	0	3	0	2
36	Unprotected	-6.49955	-68.7866	14.9	1.7	168.4	0	2	3	19
37	Unprotected	-6.51731	-68.7745	32.9	2.1	164.3	0	3	1	10
38	Unprotected	-6.49511	-68.7543	24.4	4.3	158.4	0	2	0	0
39	Unprotected	-6.49156	-68.6917	20.4	6.2	146.1	0	0	2	24
40	Unprotected	-6.4886	-68.6691	25.7	4.1	140.3	2	3	2	15
41	Unprotected	-6.47121	-68.6471	11.1	0.3	135.4	3	2	1	18
42	Unprotected	-6.50849	-68.5932	16.3	2.6	117.9	0	1	0	3
43	Unprotected	-6.46517	-68.591	23.9	0.5	128.2	0	1	0	0
44	Unprotected	-6.51294	-68.5764	11.8	3.4	115.6	0	0	0	12
45	Unprotected	-6.50064	-68.5643	19.1	3.9	113.4	0	1	0	0
46	Unprotected	-6.4731	-68.468	15.0	3.2	81.3	0	0	0	0
47	Unprotected	-6.50872	-68.451	18.8	3.9	65.8	0	0	1	2
48	CBC only	-6.49492	-68.4488	8.7	4.5	71.1	37	6	4	22
49	Unprotected	-6.47573	-68.4467	28.9	1.3	76.3	0	0	1	0
50	Unprotected	-6.49085	-68.4115	16.6	3.3	56.1	0	0	0	3

Beach ID	Protection status	Lat	Long	Area (ha)	Nearest comm. (km)	Nearest town (km)	*Rynchops niger*	*Phaetusa simplex*	*Sturnella superciliaris*	*Neochen jubata*
**51**	Unprotected	-6.50136	-68.351	48.4	5.8	39.0	0	0	0	0
**52**	Unprotected	-6.47208	-68.2735	67.0	9.4	14.6	0	0	0	0
**53**	unprotected	-6.37603	-68.1767	26.3	3.6	13.4	0	2	4	0
**54**	unprotected	-6.30559	-68.1473	21.0	6.6	33.7	0	0	0	0
**55**	unprotected	-6.3652	-68.1378	30.5	0.8	20.9	2	0	1	0
**56**	unprotected	-6.32362	-68.1363	33.0	5.5	26.9	0	2	0	0
**57**	CBC only	-6.25819	-68.1332	35.9	2.7	43.3	46	101	2	0
**58**	unprotected	-6.30943	-68.1312	12.1	7.0	31.2	0	0	0	0
**59**	unprotected	-6.20366	-68.1013	42.0	1.7	69.2	4	3	0	4
**60**	unprotected	-6.18636	-68.0847	16.6	0.5	73.4	0	4	1	2
**61**	unprotected	-6.17255	-68.0105	6.8	1.6	89.7	0	0	0	0
**62**	unprotected	-6.1831	-67.9977	13.7	0.6	91.8	0	4	0	0
**63**	unprotected	-6.12338	-67.8994	21.8	2.2	103.8	4	2	3	2
**64**	unprotected	-6.09715	-67.8721	13.6	4.3	119.7	0	0	1	0
**65**	unprotected	-6.0975	-67.8681	13.6	4.3	119.7	0	0	0	0
**66**	unprotected	-5.79165	-67.851	13.3	1.0	174.0	0	5	0	2
**67**	PA only	-5.86424	-67.8501	26.1	2.8	163.3	0	2	6	4
**68**	CBC + PA	-5.84491	-67.8435	19.2	5.0	166.1	0	11	4	0
**69**	unprotected	-6.07207	-67.8404	16.7	3.1	125.1	0	0	0	5
**70**	PA only	-5.99961	-67.8387	28.1	2.5	133.1	0	1	1	3
**71**	CBC + PA	-5.895	-67.8384	40.7	1.2	154.5	161	149	1	4
**72**	CBC + PA	-5.46848	-67.4695	33.0	1.5	251.8	241	415	0	3
**73**	CBC + PA	-6.00629	-67.8333	30.8	2.1	131.6	78	116	15	6
**74**	CBC + PA	-5.76356	-67.8302	20.4	3.1	188.7	107	55	16	4
**75**	CBC + PA	-5.80307	-67.8267	14.6	1.6	177.4	53	8	17	1
**76**	PA only	-5.92461	-67.8201	31.7	1.4	254.6	0	2	0	0
**77**	PA only	-5.90523	-67.9773	7.2	0.6	252.2	0	6	0	1

Beach ID	Protection status	Lat	Long	Area (ha)	Nearest comm. (km)	Nearest town (km)	*Rynchops niger*	*Phaetusa simplex*	*Sturnella superciliaris*	*Neochen jubata*
78	PA only	-5.79991	-67.8036	27.2	4.6	181.0	0	1	3	8
79	CBC + PA	-5.99415	-67.7989	28.8	2.5	141.4	4	8	8	4
80	PA only	-5.94511	-67.7929	27.2	2.8	147.0	16	27	0	0
81	PA only	-5.73838	-67.7775	15.6	1.2	195.8	0	4	0	0
82	CBC + PA	-5.74069	-67.7586	14.0	0.4	196.6	150	371	2	3
83	PA only	-5.60726	-67.7021	28.6	1.6	232.2	0	3	1	74
84	PA only	-5.61288	-67.678	7.2	3.2	234.4	16	13	2	38
85	PA only	-5.58966	-67.6196	6.4	5.5	255.8	0	1	1	37
86	PA only	-5.59344	-67.5964	21.5	5.8	264.9	0	0	0	47
87	PA only	-5.53698	-67.5933	25.2	1.3	275.9	0	1	1	2
88	PA only	-5.59334	-67.5772	4.9	4.7	267.1	0	2	3	0
89	PA only	-5.57234	-67.575	7.0	2.8	269.1	1	2	1	53
90	PA only	-5.55039	-67.5717	27.5	1.2	271.4	0	0	0	2
91	PA only	-5.51321	-67.5591	3.7	3.1	269.9	0	0	0	20
92	PA only	-5.52656	-67.5453	8.7	5.6	266.0	0	0	0	27
93	PA only	-5.51696	-67.4912	17.4	3.9	259.8	0	2	0	0
94	CBC + PA	-5.50004	-67.4815	34.7	2.2	257.5	8	14	0	0
95	PA only	-5.48616	-67.4417	24.2	4.1	247.5	0	0	0	24
96	PA only	-5.37749	-67.3976	52.9	0.6	222.9	0	6	5	2
97	CBC + PA	-5.41702	-67.3744	122.9	3.8	214.2	18	12	5	0
98	CBC only	-5.4142	-67.3023	32.0	1.1	202.7	198	262	6	0
99	PA only	-5.37893	-67.2408	46.2	4.0	192.2	0	1	0	0
100	CBC + PA	-5.19178	-67.2291	32.9	9.6	139.6	151	270	1	0
101	PA only	-5.17365	-67.2067	30.9	7.3	145.7	0	2	1	1
102	PA only	-5.16692	-67.188	29.8	9.0	142.2	0	2	1	4
103	PA only	-5.08093	-67.1363	27.8	1.1	110.1	0	1	7	2
104	CBC + PA	-5.14466	-67.1193	51.3	6.0	123.0	270	379	0	0

Beach ID	Protection status	Lat	Long	Area (ha)	Nearest comm. (km)	Nearest town (km)	*Rynchops niger*	*Phaetusa simplex*	*Sturnella superciliaris*	*Neochen jubata*
105	CBC + PA	-5.0871	-67.1161	48.1	4.2	105.4	302	162	0	0
106	PA only	-5.10243	-67.082	16.5	5.2	95.4	0	1	1	0
107	unprotected	-5.07573	-67.0109	22.2	1.4	84.0	0	6	4	0
108	unprotected	-5.07228	-66.9915	15.4	4.0	80.8	0	2	0	2
109	unprotected	-5.06338	-66.9768	6.8	5.6	79.1	0	0	0	0
110	unprotected	-5.05156	-66.9587	42.6	4.6	76.4	0	0	1	0
111	unprotected	-5.04286	-66.952	25.2	3.6	74.1	0	0	0	0
112	unprotected	-5.04923	-66.9427	0.5	2.5	72.8	0	0	0	0
113	unprotected	-5.07659	-66.931	46.6	1.9	68.8	0	0	0	0
114	unprotected	-5.03684	-66.8992	29.8	3.7	58.1	0	0	0	0
115	unprotected	-5.00734	-66.8773	9.9	8.3	52.5	0	0	0	0
116	unprotected	-4.99468	-66.8659	19.2	11.0	29.8	0	1	13	0
117	unprotected	-4.94818	-66.8583	53.5	8.8	24.7	0	2	2	2
118	unprotected	-4.84084	-66.7761	32.7	7.4	10.8	0	2	4	0
119	unprotected	-4.79591	-66.7749	28.5	7.7	16.8	0	3	0	1
120	unprotected	-4.82458	-66.7611	9.9	9.3	13.1	0	0	0	0
121	unprotected	-4.74096	-66.7407	16.7	1.2	32.0	0	1	2	2
122	unprotected	-4.76436	-66.73	57.5	4.6	27.8	0	0	2	0
123	unprotected	-4.62624	-66.676	31.6	5.7	58.2	4	10	8	0
124	unprotected	-4.69683	-66.6624	19.1	1.1	48.0	0	4	4	2
125	unprotected	-4.57248	-66.6604	19.9	3.0	69.3	1	3	7	0
126	unprotected	-4.58335	-66.6544	17.2	1.6	67.5	0	3	1	0
127	unprotected	-4.6704	-66.6459	24.1	1.5	52.0	0	1	2	0
128	unprotected	-4.61462	-66.6399	40.8	2.8	63.0	1	2	32	0
129	unprotected	-4.559	-66.6352	20.7	2.4	73.8	0	0	0	0
130	unprotected	-4.45984	-66.5863	16.2	7.5	89.8	2	0	2	0
131	unprotected	-4.50688	-66.5666	18.8	3.4	83.4	0	0	4	0

Beach ID	Protection status	Lat	Long	Area (ha)	Nearest comm. (km)	Nearest town (km)	*Rynchops niger*	*Phaetusa simplex*	*Sturnella superciliaris*	*Neochen jubata*
132	unprotected	-4.45579	-66.5628	8.0	6.4	92.3	0	0	0	0
133	unprotected	-4.36134	-66.5622	18.0	1.4	119.1	0	1	0	0
134	unprotected	-4.40268	-66.5403	30.7	3.9	108.8	4	6	9	0
135	unprotected	-4.35739	-66.5371	14.9	1.5	122.1	2	2	3	0
136	unprotected	-4.33145	-66.5242	17.5	4.6	125.6	1	2	2	0
137	unprotected	-4.30895	-66.52	10.3	6.1	131.8	1	0	0	3
138	unprotected	-4.33631	-66.4992	10.0	5.7	129.1	1	1	0	0
139	unprotected	-4.12051	-66.4579	18.0	5.1	173.5	0	1	3	0
140	CBC only	-3.81303	-66.3252	33.0	5.7	148.8	346	221	23	0
141	unprotected	-3.82539	-66.2987	9.7	0.8	131.4	0	1	1	0
142	unprotected	-3.81219	-66.2728	5.3	1.8	127.5	0	0	2	0
143	unprotected	-3.73311	-66.2465	4.4	6.4	106.0	0	0	0	0
144	unprotected	-3.74589	-66.2242	4.3	5.2	109.1	3	1	2	0
145	unprotected	-3.7095	-66.2238	11.8	2.2	89.4	9	2	0	0
146	unprotected	-3.67814	-66.1945	11.6	2.2	78.8	0	3	1	0
147	unprotected	-3.75233	-66.1865	4.7	5.1	96.6	0	2	1	0
148	unprotected	-3.69967	-66.1752	12.0	2.8	82.9	1	0	0	0
149	PA only	-3.647	-66.1507	39.5	3.8	66.7	0	3	18	0
150	unprotected	-3.49125	-66.1341	10.0	3.0	17.7	0	0	1	0
151	CBC only	-3.59964	-66.1002	15.1	3.6	50.9	43	25	13	0
152	unprotected	-3.50381	-66.0884	18.9	1.1	25.9	1	1	1	0
153	PA only	-3.60425	-66.0844	10.9	0.7	47.3	0	3	1	0
154	unprotected	-3.46264	-66.0835	26.5	2.9	3.5	0	0	0	0
155	CBC + PA	-3.20434	-65.9766	52.9	1.7	51.0	178	211	47	0
