# Supplementary material for: Community-based conservation with formal protection provides large collateral benefits to Amazonian migratory waterbirds
Source: PLoS One. 2021 Apr 8;16(4):e0250022. doi: 10.1371/journal.pone.0250022 (PMC8031428; doi:10.1371/journal.pone.0250022)
Supplement: S2 Table — Summary of GLM analysis showing the top models (ΔAICc < 2) for each species. (PDF) [file pone.0250022.s005.pdf]

S2 Table. Model selection explaining the abundance of waterbirds on fluvial beaches along the Juruá River, western Brazilian Amazonia. Summary of GLM analysis showing the top models (ΔAICc < 2) for each species.

Species	Models	df	logLik	AICc	delta	weight
Rynchops niger	*Distance to town + Distance to community + Protection status*	5	98.07	-185.7	0	0.427
	Distance to town + Protection status	6	98.753	-184.9	0.80	0.287
	Distance to community + Protection status	6	98.243	-183.9	1.82	0.172
Phaetusa simplex	*Distance to community + Protection status*	6	65.57	-118.6	0	0.572
Sternulla superciliaris	*Protection status*	5	203.6	-397.0	0	0.479
	Distance to town + Protection status	6	204.1	-395.7	1.27	0.253
Neochen jubata	*Distance to town + Protection status*	6	73.9	-135.3	0	0.671
	Distance to town + Distance to community +Protection status	6	74.09	-133.4	1.85	0.267
