# Supplementary material for: Community-based conservation with formal protection provides large collateral benefits to Amazonian migratory waterbirds
Source: PLoS One. 2021 Apr 8;16(4):e0250022. doi: 10.1371/journal.pone.0250022 (PMC8031428; doi:10.1371/journal.pone.0250022)
Supplement: S1 Methods — (PDF) [file pone.0250022.s006.pdf]

S1 Methods. Protocol for the community-based protection of freshwater turtles by beach guards

The community-based management of Amazonian freshwater turtles targets the protection of beaches and breeding females along major rivers within the turtle reproductive season (Balestra, 2016; Campos-Silva et al. 2020). In this context, beach guards from local communities have two main duties: (i) the prevention of poaching, and (ii) the monitoring of nesting success.

Beach guards conduct surveillance of nest sites to prevent the activity of poachers, precluding the illegal harvesting of eggs and female turtles. They also serve to prohibit the presence of people and boats, including fishers and fishing boats, on or near nesting beaches. This is to ensure that turtles are able to spawn on the beach and return to the river or lake without disturbance after spawning. Beach guards continue to maintain a permanent presence at nesting beaches until hatchling emergence is completed, whilst conducting population monitoring activities. Beach guards monitor oviposition by nesting female turtles and the subsequent emergence of hatchlings. This is achieved by recording the date and location of each nest, calculating the expected date of hatching and, on the date of emergence, recording the number of eggs and successful hatchlings.
