# Supplementary material for: Community-based conservation with formal protection provides large collateral benefits to Amazonian migratory waterbirds
Source: PLoS One. 2021 Apr 8;16(4):e0250022. doi: 10.1371/journal.pone.0250022 (PMC8031428; doi:10.1371/journal.pone.0250022)
Supplement: Attachment — Submitted filename: Point_by_point_responses_final.docx [file pone.0250022.s007.docx]

**Journal:** PLOS ONE

**Ref:** PONE-D-20-19231

**Title:** Community-based conservation with formal protection provides large collateral benefits to migratory waterbirds of Amazonia

**Point by point review response**

Journal Requirements:

When submitting your revision, we need you to address these additional requirements.

1. Please ensure that your manuscript meets PLOS ONE's style requirements, including those for file naming.

**Authors:** We have made sure that we are now following all the style requirements, including file naming.

2. In your Methods section, please provide additional location information of the study sites, including geographic coordinates for the data set if available.

**Authors:** We included the geographic coordinates of our study area in line 121. In addition, we provided the geographic locations of all sampled beaches in the Supporting Information section.

3. We note that you have provided funding information that is not currently declared in your Funding Statement. However, funding information should not appear in the Acknowledgments section or other areas of your manuscript. We will only publish funding information present in the Funding Statement section of the online submission form.

Please remove any funding-related text from the manuscript and let us know how you would like to update your Funding Statement. Currently, your Funding Statement reads as follows: [The funders had no role in study design, data collection and analysis, decision to publish, or preparation of the manuscript.]

**Authors:** We removed this funding information from the Acknowledgements and reinserted all funding information into the Funding Statement. We would like our Funding Statement to be modified to the following:

“This study was funded by a Darwin Initiative for the Survival of Species grant (DEFRA, UK; Ref. 20-001) to C.A.P., a CAPES PhD scholarship (Ref. 1144985) and CAPES postdoctoral grant (Ref. 1666302) to J.V.C.S., a CAPES postdoctoral grant (Ref. 1530532) and internal funding from Anglia Ruskin University to J.E.H., and a National Geographic Society grant (Ref. WW1-277R-18) to L.D. The funders had no role in study design, data collection and analysis, decision to publish, or preparation of the manuscript. J.V.C-S acknowledge the Research Council of Norway for funding his post-doc position (grant no. 295650).”

4. We note that [Figure(s) 1 and 4] in your submission contain [map/satellite] images which may be copyrighted. All PLOS content is published under the Creative Commons Attribution License (CC BY 4.0), which means that the manuscript, images, and Supporting Information files will be freely available online, and any third party is permitted to access, download, copy, distribute, and use these materials in any way, even commercially, with proper attribution. For these reasons, we cannot publish previously copyrighted maps or satellite images created using proprietary data, such as Google software (Google Maps, Street View, and Earth). For more information, see our copyright guidelines: <http://journals.plos.org/plosone/s/licenses-and-copyright>. We require you to either (1) present written permission from the copyright holder to publish these figures specifically under the CC BY 4.0 license, or (2) remove the figures from your submission.

**Authors:** Our maps were produced using Landsat data (https://landsat.usgs.gov/), freely available from the USGS archive (https://www.usgs.gov/centers/eros/data-citation). We have now included this information in the figure legends.

5. Please include captions for your Supporting Information files at the end of your manuscript, and update any in-text citations to match accordingly. Please see our Supporting Information guidelines for more information: http://journals.plos.org/plosone/s/supporting-information.

**Authors:** We have now included captions for the Supporting Information.

**Reviewer 1:** I read this manuscript with great interest. The topic is very worthy of study given that the Amazon is a major biodiversity hotspot yet the effectiveness of different types of protected areas has received limited attention. At face value the conclusions offer valuable new information on how community-led conservation efforts can have major benefits for wildlife, not only for the target species (in the case of CBCs, freshwater turtles), but also waterbird species which were the focus of your study. That said, the value of conservation research is dependent on appropriate study design and analyses. Having read your study, I had a number of concerns about the data collection and analyses:

**Authors:** Thank-you very much for your very constructive review. We appreciate all your comments and suggestions that have strengthened the manuscript, and we hope that it is now suitable for publication in PLOS ONE.

**Reviewer 1:**  (i) I was not clear on why the statistical analyses are done in two steps, the first which looks just at the protected area designation, and the second which attempts to account for covariate effects. I do not see why these could not have been combined into a single analysis that tested for differences in abundance between different protected area designations whilst accounting for key covariates such as beach area. A single analysis would have been optimal. The first analysis in particular, did not appear to test the assumptions correctly (normality is assumed in the residuals, not the raw data, and the other assumptions also need to be assessed), whilst not accounting for factors such as beach area which could have affected bird counts. In my view the first analysis could be dropped and the analyses refocused on the second analysis.

**Authors:** We agree with the reviewer. We have now combined the analysis into a single generalized linear model for each species as recommended, and we believe that the results are much clearer now. Details on our new analytical approach can be found from lines 181 to 217 within the Methods section.

(ii) There is likely to be an issue with spatial autocorrelation in your data set, that needs to be accounted for within your analysis. Figure 1 shows clearly that many of your sites of each designation type (e.g. CBC) are clustered together. This is often inevitable in real-world conservation situations, but it presents a problem when analyzing such data because sites that are closer together are more likely to have similar waterbird abundances than sites which are further apart. They are also more likely to be more similar in terms of their covariate values. As a first step, you should use a metric such as Moran's I to quantify the strength of spatial autocorrealtion in your data set. There are a number of R packages that allow you to estimate Moran's I and test its significance, such as the spdep package. If spatial autocorrelation is very weak (i.e. non-significant) you may be ok with your original analysis, but if it is significant you will need to account for the autocorrelation in order to achieve accurate and robust conclusions. The following two papers provide examples of how this could be achieved: Beale CM, Lennon JJ, Yearsley JM, Brewer MJ, Elston DA. 2010. Regression analysis of spatial data. Ecology Letters 13:246-264. Dormann CF, McPherson JM, Araújo MB, Bivand R, Bolliger J, Carl G, Davies RG, Hirzel A, Jetz W, Kissling WD. 2007. Methods to account for spatial autocorrelation in the analysis of species distributional data: a review. Ecography 30:609-628.

**Authors:** Thank you very much for your recommendation. is the Reviewer correctly observed a cluster of community-protected beaches in the central part of our study area, partly due to a stronger governance model in this area which stimulates local communities to protect their own territories. We have therefore performed the Moran's I test as suggested and can confirm that the result of this test does not show a significant degree of spatial autocorrelation. We report the results of this analysis in lines 184-194 in the Results. Since this effect was not significant, we then resumed our original analysis without incorporating spatial autocorrelation in our models, as explained in lines 191-194.

(iii) There needs to be more explicit consideration of the process of designation of the different types of protected area status on the beaches. Currently, the manuscript attempts to link the higher abundances in certain types of site (e.g. CBC) with the protection status of the site. But could it be the case that these areas already had higher abundances of waterbirds when they were designated, rather than having achieved higher abundances because of the status? Especially if the sites were chosen because they were high quality sites for turtles, could it be that they were also inherently high quality sites for waterbirds? Globally, it is frequently the case that sites of high abundance are designated as protected areas for that species, which complicates assessments of the effects of protected areas. At the very least, your manuscript ought to discuss this issue and clarify what you think the mechanisms are through which CBC sites come to have higher waterbird abundances, and what the evidence is for these mechanisms (keeping in mind that in this paper you report only waterbird abundance estimates taken some time after CBC designation).

**Authors:** We thank the reviewer for this important point, as we recognize that this is a general phenomenon that needs to be considered. However, we do not feel that this issue is of particular influence in this situation. Firstly, most protected beaches came under protection within the last decade and some of them became protected only two years ago. We now clarify this point in lines 159-165. Secondly, local beach guards often support the view that local protection is the strongest factor determining the population size of waterbirds, reporting that bird populations were small to very small in beaches prior to the onset of local beach protection, and that bird abundance noticeably increased over the years after protection. By plotting the population size of birds as a function of the number of years, we can confirm this positive relationship, especially for beach-dependent species such as *Rynchops niger* and *Phaetsua simplex* (Figure A, below). We have now included this point in our discussion of the potential mechanisms operating at community-protected beaches in lines 326-239, and include this figure in the Supporting Information (Fig. S3).

**Figure A.** Population size of target species (a) *Rynchops niger*, R^2^ = 0.8, p<0.01; (b) *Phaetusa simplex*, R^2^ = 0.6, p<0.01; (c) *Sturnella superciliaris*, R^2^ = 0.1, p<0.01; and (d) *Neochen jubata,* R^2^=-0.003, p=0.4) as a function of the number of years that the beach had been under local community protection for unprotected sites (red), PA-only (blue), CBC-only (orange) and CBC + PA (yellow). Unprotected sites and PA-only were left as zero years of protection, considering that in both categories there is no local site protection by rural communities.

(iv) I also had some concerns about the waterbird survey methodologies. I appreciate that surveying large areas in difficult terrain is logistically challenging, but some consideration needs to be given to the issue of birds that could have been missed or double-counted. This is because the surveys of the 155 beaches were not synchronous; given the length of the study areas and the movement speed that you report, the surveys were undertaken over the course of many days (it would be useful for you to clarify the exact time period in August 2016, in terms of numbers of days). Given what is known about the 4 species, it is possible that individuals moved between sites over the course of your surveys (especially if the surveys disturbed birds from the beaches), resulting in birds that were either missed or double-counted? As your study was carried out in the breeding season, the birds may have been largely site-faithful, but given the close proximity of many of your sites, I think that at the least you should mention these issues in either the methods or discussion, along with your thoughts on whether these issues could have affected your count data.

**Authors:** We agree that a multi-year approach could better elucidate the variation in population size of our target species, but unfortunately, it was not possible to repeat the survey due to the scale of our sampling design, which covered a fluvial distance of ~1,600 km along the Juruá River. Even recognizing the limitation of our methods, we are confident that we are capturing the wider patterns of bird abundance. For example, we compared though paired t test the population size for two bird species from a subset of 28 beaches that were surveyed in 2014 (Campos-Silva et al. 2018) and in 2018 (for this current manuscript), and we found no difference between years (Figure B, below). We therefore believe that, even with a potential error regarding the real number of individuals, we can have a good estimate to investigate the population size across different classes of protection. We appreciate the reviewer's concern, and we have included a sentence in our Discussion to ensure transparency of any limitation in our methods (lines 329-336), and include this figure in the Supporting Information (Fig. S2).

**Figure B**. We used a subset of 28 beaches to compare our surveys conducted in 2016 (for this current manuscript) with the surveys conducted in 2014 (Campos-Silva et al. 2018). We present here the consistent population size of (a) *Rynchops niger* and (b) *Phaetusa simplex* at “CBC + PA” and “PA-only” beaches in each of these surveys.

(v) My other concern about the waterbird survey methodology was the lack of standardization of the timing of the surveys. For diurnally-active bird species, activity patterns can show consistent patterns across daylight hours (for example, see: Robbins, C.S. 1981. Effect of time of day on bird activity. Studies in avian biology, 6, 275-286). The fluctuations in activity patterns can result in detection probabilities that vary with time of day (Palmeirim, J.M. & Rabaça, J.E. 1994. A Method to Analyze and Compensate for Time-of-Day Effects on Bird Counts. Journal of Field Ornithology, 65, 17-26). Therefore, in your study the abundance of birds on a beach could have been affected by the time during the day that the count was conducted. As your counts started early in the day, could this have included some individuals that would have subsequently left their nest area to forage elsewhere, i.e. would these early counts have recorded lower abundance estimates if they'd been made in the middle of the day? If you have recorded the time of each survey, you could account for time-of-day (perhaps in blocks of time, e.g. 06:30-09:30, 09:30-12:30, and so on) within your models.

**Authors:** Thank you very much for your concern. We are aware that time of day frequently has a substantial effect on avian behavior. Our census walks started at 06:30h and did not finish later than 10:00h. We have now clarified this in the Methods (lines 170-173). To verify that our results were not influenced by time of the day, we analyzed our data considering time of day (Figure C, below). As we did not find any significant difference between early morning and later morning surveys, we did not include this factor in our subsequent models. Since most of our target waterbird species use the beaches to breed, rather than for other activities (e.g. foraging), we believe that our surveys provide a robust estimate of breeding bird population sizes on that beach. We clarified this the Methods (lines 203-206), and we also included this figure in the Supporting Information (Fig. S1).

**Figure C**. Investigation of the effect of time of the day in our survey. Time 1 and 2 represents surveys conducted from 06:30h to 08:00h and from 08:30h to 10:00h, respectively. The target species are represented by (a) *Rynchops niger*, (b) *Phaetusa simplex*, (c) *Sturnella superciliaris,* and d) *Neochen jubata.*

I also have a number of additional, more specific comments, which I hope will help you to improve your study:

Page 3, Lines 44-46: Here, I recommend also adding the following classic paper on tropical freshwater ecosystems, which highlights many of the issues that these ecosystems face: Dudgeon, D. (1992). Endangered ecosystems: a review of the conservation status of tropical Asian rivers. Hydrobiologia, 248, 167-191.

**Authors:** Thank you very much for your suggestion. We have included a citation to this paper.

Page 3, Lines 56-62: Do the CBCs allow some limited exploitation of turtles by local communities, or is no exploitation allowed? The information in the next paragraph suggests that the latter is true, but it would be good to clarify this point to help the reader understand how the system works.

**Authors:** Yes, we can confirm that no exploitation of turtles is allowed at these protected beaches. We have clarified this in lines 59-60.

Page 5, Line 105: The "-" is not needed before "km" and so can be deleted.

**Authors:** Done.

Page 8, Line 168: Do you mean 20 birds in total (of all species) or 20 individuals of a single species?

**Authors:** When a bird colony was more than 20 individuals in total (all species combined), the observer repeated the count three times, using the smallest number of individuals as a conservative estimate. Large flocks mostly consisted of one species, generally *Rynchops niger* or *Phaetusa simplex*. We clarified this information in lines 173-176.

Page 8, Lines 179-182: Was Kruskal-Wallis used here as a non-parametric alternative to ANOVA? Crucially, ANOVA assumes normality of the residuals, not the raw data, so what you've done here is not the correct procedure. You also have not mentioned any other test assumptions beyond normality. ANOVA is a more powerful test than Kruskal-Wallis and ought to be used if the asssumptions are met.

**Authors:** This section has been removed following our updated analysis.

Page 8, Line 180: Typo -> "Kruskal" not "Kruskall".

**Authors:** This section has been removed following our updated analysis.

Page 9, Line 203: More detail is needed here. Specifically, which assumptions did you check and how did you do this? Did you use visual inspections of residuals or formal statistical tests? Also, how did you test for collinearity? Did you use Variance Inflation Factors or correlations? These results should be reported in the manuscript.

**Authors:** This section has been removed following our updated analysis. We also provided more details regarding the assumptions of our analysis in lines 215-217.

Page 12, Line 245: Do the P values in the table (and elsewhere in the manuscript) take account of the fact that multiple p values have been calculated? Using Holm-Bonferroni corrections (or similar) would resolve this issue.

**Authors:** This section has been removed following our updated analysis.

Page 12, Lines 254-256: The results reported here do not appear to match the values reported in Table 1. For example, the text states that "mean population size of Black Skimmer was 135-fold and 108-fold larger on CBC only and CBC + PA beaches than at unprotected sites, and only 1.3 higher in PA only beaches compared with unprotected sites", but the data in Table 1 show that population size on CBC only beaches is 134 vs 1 at unprotected sites, 107.2 at CBC + PA sites, and 1.2 at PA only versus 1.0 at unprotected sites. I recommend checking and revising the entire results section so that the values reported in the text agree with the values reported in the tables and figures.

**Authors:** The results were carefully checked after our updated analysis.

Page 15, Line 305: As per major comment iii above, I think that there needs to be a more nuanced interpretation of your results. You do not have time series data to show how waterbird abundances have changes pre- and post-designation as a CBC site (or as any other type of protected area) so you cannot conclude that species have "responded" to site protection. As mentioned earlier, it may be the case that these sites already had high waterbird abundances when they were designated as CBC sites, or it could be that the protection afforded to CBC sites has resulted in the higher abundances. Unless you have the data to distinguish between these two hypotheses, you need to be more cautious in how your frame your findings.

**Authors**: We agree with the reviewer, and we reframed our findings to avoid using terms like ‘responded’ that would imply an analysis of time series data.

Page 16, Lines 346-347: Can you cite any evidence to support the idea that smaller beaches have lower fishing activity?

**Authors**: This section has been removed following our updated analysis.

Page 25, Lines 532-534: Which version number of R did you use in your analyses? Please add that information here.

**Authors:** We have now included the version number in this citation (line 217).

Reviewer #2: In this paper, Campos-Silva et al monitor the population of four waterbirds on 155 beaches along the Juruá River and find that the waterbirds populations are significantly larger on the CBC beaches than that on the other beaches. Therefore, Campos-Silva et al declare that the collateral benefits from local community’s conservation activities on turtles explain why the four waterbirds aggregate on the CBC beaches using regression analysis. This is an important area of biodiversity conservation research, particularly in a biodiversity hotspot region with high level of human disturbance.

**Authors:** Thank you very much for your very constructive review. We appreciate all your comments and suggestions which have undoubtedly strengthened the manuscript, and we hope that it is now suitable for publication in PLOS ONE.

Reviewer #2: My first concern is the authors monitor the waterbirds population only one time. Due to the strong ability and large range of birds flying, it is doubtful whether only one survey can truly reflect the actual population numbers of the four birds.

**Authors:** This was also a concern from Reviewer 1. Please see our response to this point above.

My second concern is that the collateral benefits from community conservation is not fully proven in the manuscript. Bird population is affected by a variety of environmental factors, including temperature, precipitation, food resources, etc., which have been confirmed in a series of literatures. In lines 354-355, the authors quoted their previous research to show that community conservation overshadow other environmental factors, which is difficult to convince readers. Figure 4 shows that most of the beaches where waterbirds congregate are distributed in the middle of the Juruá River. Therefore, it is also possible that the longitude, latitude, temperature or food resources determine the bird population, while CBC happens to be run in the suitable beaches for waterbirds. Furthermore, the high SD values of Black Skimmer and Large-Billed Tern on the PA only beaches somewhat demonstrate that some environmental factors other than CBC mainly influence waterbirds populations. Therefore, it is necessary for the author to take more environmental parameters into account when construct the regression models to verify the conclusion. If the strengthened model still shows that CBC is the dominant factor, the paper will be more convincing. If the conclusion of the strengthened model is not consistent with the current one, the manuscript needs to make corresponding modifications before publication.

**Authors:** We agree with the reviewer that waterbirds can show strong nesting beach preferences in relation to environmental factors other than level of protection, including beach slope and substrate quality. However, we also believe that the balance between human and environmental factors is already well described in Campos-Silva et al. (2018). In this previous study, we showed that human factors such as beach protection are so strong that they completely override any environmental covariate, which is largely consistent across the longitude, latitude, and climatic ranges within the study area. Moreover, to further demonstrate this, the environmental factors at a subset of 28 beaches shows that there are no significant differences in physical factors between protected and unprotected beaches (Figure D, below). Therefore, in this current study, we focused our efforts mostly on human factors such as protected areas and community-based conservation, to understand the main responses of waterbirds to human governance on a large scale within the same study region as that assessed by Campos-Silva et al. (2018). However, we recognize the importance of environmental factors and we have emphasized this point in our Discussion in lines 355-362.

**Figure D.** Paired physical characteristics of beaches along the Juruá River, showing no significant differences in (A) beach slope; (B) beach arc length; (C) beach area and (D) % coarse sand. Yellow and purple boxplots represent protected (PB) and unprotected beaches (UB), respectively. This figure is already published in the Supplementary Information of Campos-Silva et al. (2018).
