# Supplementary material for: Community-based conservation with formal protection provides large collateral benefits to Amazonian migratory waterbirds
Source: PLoS One. 2021 Apr 8;16(4):e0250022. doi: 10.1371/journal.pone.0250022 (PMC8031428; doi:10.1371/journal.pone.0250022)
Supplement: Attachment — Submitted filename: Campos_PLOSONE_2021.docx [file pone.0250022.s008.docx]

Campos-Silva et al. provide compelling evidence that river areas protected local communities for turtle nesting provide additional benefits for colonial nesting waterbirds, but not for a solitary foraging goose species. The authors compared and surveyed three areas: unprotected, government designated protected areas (PAs) and community-based conservation (CBC) areas in the Amazon. The spatial coverage was significant: 1600 km and 155 sites. The analyses appeared to address the issue of many sites that had no nesting waterbirds (i.e., zeros). What is interesting about this study is the findings that CBC programs, implemented for a different taxon (turtles), had an umbrella positive benefit for all three species of colonial nesting waterbirds, but not for the Orinoco goose. The analyses made sense and their conclusions, that CBC programs can have added benefits to other species, were based on a strong study design. While there is recent discussion and push to involve local communities in conservation initiatives, there are few data that evaluate that management option, thus this study is a valuable contribution.

Specific comments

Line 69 beach guards (CBC) – Is there a formal protocol that the beach guards follow beyond what was described in the Introduction? If yes, could it be shared under Supplemental section? I wonder if for example, they are permitted to have dogs, or if dogs are prohibited? The presence of dogs would presumably negatively impact ground nesting waterbirds.

Line 161 waterbird surveys - Were birds flushed during the survey? If yes, is it possible they relocated to another area being surveyed? (i.e., confirm no double counting within surveys happening simultaneously).

Line 209 Results - While the beach area for each survey site is presented in the supplemental data, it would be good to provide some descriptive stats (area per category) to get a sense of the distribution of beach area for Unprotected, PA and CBC.
