# Supplementary material for: Community-based conservation with formal protection provides large collateral benefits to Amazonian migratory waterbirds
Source: PLoS One. 2021 Apr 8;16(4):e0250022. doi: 10.1371/journal.pone.0250022 (PMC8031428; doi:10.1371/journal.pone.0250022)
Supplement: Attachment — Submitted filename: Point_by_point_responses_final.docx [file pone.0250022.s009.docx]

**Journal:** PLOS ONE

**Ref:** PONE-D-20-19231

**Title:** Community-based conservation with formal protection provides large collateral benefits to migratory waterbirds of Amazonia

**Point by point review responses**

Reviewer #1: Thank you for the thorough and well-documented revisions that you have carried out. I think that you have addressed all of our points and the manuscript has been greatly improved. Reading through your revised text, I had a few final comments and suggestions, which I hope will prove useful.

Authors: We appreciate the reviewer’s effort in improving our manuscript, and are grateful for all their comments and suggestions.

Lines 48-50. I agree, the region has such high biodiversity value but faces a range of anthropogenic threats. In addition to the reference you already give, I would recommend also citing this subsequent paper which provides the latest perspective on this topic: Castello, L. & Macedo, M.N. (2016). Large‐scale degradation of Amazonian freshwater ecosystems. Global Change Biology, 22, 990-1007.

Authors: Done. Citation added to line 48.

Line 51. Can you clarify the phrase “most comprehensive protected area systems” to explain in what way(s) the protected area system is comprehensive? In terms of area? Number of sites? Number of restrictions on anthropogenic activities? Or something else?

Authors: The Brazilian protected area (PA) system is composed of 2201 PAs covering more than 250 million hectares. We clarified this point in lines 51-52.

Lines 87-88. “Increasing and strengthening conservation strategies both inside and outside PA boundaries is therefore imperative to maintaining migratory bird populations.” I think that this idea, which is fundamental to the rationale of your study, should be developed further before the readers moves to the next paragraph. Specifically, I think that you should highlight the need for waterbird monitoring, as that it critical to efforts to diagnose and alleviate anthropogenic threats to waterbird populations. Without monitoring of waterbird populations, we would lack the information necessary to undertake protective measures, such as designating protected areas or regulating anthropogenic activities that impact populations. here are two recent examples that illustrate the point: Wood, K.A., Brown, M.J., Cromie, R.L., Hilton, G.M., Mackenzie, C., Newth, J.L., Pain, D.J., Perrins, C.M. & Rees, E.C. (2019). Regulation of lead fishing weights results in mute swan population recovery. Biological Conservation, 230, 67-74. Martínez-Abraín, A., Jiménez, J., Gómez, J.A. & Oro, D. (2016). Differential waterbird population dynamics after long-term protection: The influence of diet and habitat type. Ardeola, 63, 79-101. I think that these few extra lines and references would help to steer the reader towards appreciating why waterbird monitoring is needed and what could be gained from this information, which would give the reader a greater appreciation for your study.

Authors: Thank you very much for your suggestion. We have now highlighted in the text the need for strengthened monitoring of waterbird populations (lines 88-93), and included the suggested references.

Lines 394-397. “This challenge should be shared more widely with organizations focused on bird conservation, since the collateral effects from a freshwater turtle CBC are now shown to play a central role in the conservation of waterbirds at a critical reproductive stage of their lifecycle.” Can you develop your recommendation further here, for example by giving recommendations for how the challenge could be shared effectively? Conservation projects with broader suits of biodiversity and social development targets, with stakeholders from across a more diverse range of stakeholders? Monitoring programmes undertaken by multiple stakeholder groups?

Authors: Authors: We have developed our recommendation section within the Conclusion, as suggested (lines 402 to 409).

Reviewer #3: Campos-Silva et al. provide compelling evidence that river areas protected local communities for turtle nesting provide additional benefits for colonial nesting waterbirds, but not for a solitary foraging goose species. The authors compared and surveyed three areas: unprotected, government designated protected areas (PAs) and community-based conservation (CBC) areas in the Amazon. The spatial coverage was significant: 1600 km and 155 sites. The analyses appeared to address the issue of many sites that had no nesting waterbirds (i.e., zeros). What is interesting about this study is the findings that CBC programs, implemented for a different taxon (turtles), had an umbrella positive benefit for all three species of colonial nesting waterbirds, but not for the Orinoco goose. The analyses made sense and their conclusions, that CBC programs can have added benefits to other species, were based on a strong study design. While there is recent discussion and push to involve local communities in conservation initiatives, there are few data that evaluate that management option, thus this study is a valuable contribution.

Authors: We are grateful for Rev#3’s very positive comments on our manuscript, including its novelty value in highlighting rare evidence of the conservation co-benefits accrued to non-target species.

Specific comments
Line 69 beach guards (CBC) – Is there a formal protocol that the beach guards follow beyond what was described in the Introduction? If yes, could it be shared under Supplemental section? I wonder if for example, they are permitted to have dogs, or if dogs are prohibited? The presence of dogs would presumably negatively impact ground nesting waterbirds.

Authors: We have included the main obligations of beach guards in the Supporting Information (S1 Methods). Although there is no direct mention regarding the presence of domestic and feral dogs in protected beaches, their access to beaches is informally banned because they also represent a clear depredation threat for nesting turtles, as well as waterbirds.

Line 161 waterbird surveys - Were birds flushed during the survey? If yes, is it possible they relocated to another area being surveyed? (i.e., confirm no double counting within surveys happening simultaneously).

Authors: Waterbirds are habituated to the presence of beach guards who conducted surveillance of the beaches on foot on a daily basis. Our sampling was conducted at a distance, so our presence did not scare the birds away. In addition, since most of our target waterbird species use the beaches to breed and exhibit a great deal of nest-site fidelity, rather than for other activities (e.g. foraging), we are confident that our surveys provide a robust estimate of breeding bird population sizes on each beach. We clarified this point in the Methods (lines 183-185).

Line 209 Results - While the beach area for each survey site is presented in the supplemental data, it would be good to provide some descriptive stats (area per category) to get a sense of the distribution of beach area for Unprotected, PA and CBC.

Authors: Thank-you. We agree with this suggestion and have inserted the mean area and standard deviation for each category of beach protection in the first paragraph of our Results (lines 228-231).
